# Development and evaluation of the pharmacist intervention evaluation (PIE) system: a modified classification for drug-related problems in Chinese tertiary hospitals

**DOI:** 10.3389/fphar.2026.1808669

**Published:** 2026-03-19

**Authors:** Chunlei Li, Jinyu Cui, Xiao Liang, Lingzi Zhang, Xiaoyi Guo, Yanxiao Xiang, Anchang Liu

**Affiliations:** 1 Department of Pharmacy, Qilu Hospital of Shandong University, Jinan, China; 2 Department of Clinical Pharmacy, School of Pharmaceutical Sciences, Shandong University, Jinan, China; 3 Department of Pharmacy, Qilu Hospital (Qingdao), Cheeloo College of Medicine, Shandong University, Qingdao, China

**Keywords:** China tertiary hospitals, classification efficacy, drug-related problems (DRPs), pharmaceutical care network Europe (PCNE), pharmacist intervention evaluation

## Abstract

**Background:**

Identification and intervention of drug-related problems (DRPs) is a cornerstone for pharmaceutical care. An effective classification system of DRPs enables to identify the origin of DRPs and improve performance management of pharmacists. However, a standardized DRPs classification system tailored to the Chinese healthcare environment has not been established.

**Aim:**

To establish and validate Pharmacist Intervention Evaluation (PIE) System, which is a modified Pharmaceutical Care Network Europe (PCNE) DRPs classification system, and further compared the efficacy, acceptability, and feasibility with PCNE system in tertiary hospitals in China.

**Methods:**

This study used a multi-phase methodological design comprising (1) development and content refinement of the PIE classification through structured expert consensus and pilot testing, and (2) a prospective, multi-center field evaluation using consecutively captured DRP reports from routine clinical pharmacy practice. A total of 1398 DRP reports were identified, intervened, and recorded by clinical pharmacists according to PCNE and PIE systems, respectively. The classification efficacy, acceptability, and feasibility of the two systems were compared. Multivariate regression analysis was employed to identify factors associated with classification efficacy of PIE system.

**Results:**

PIE system, consisting of 6 primary and 27 secondary categories, was constructed and validated through expert consensus and multi-center prospective studies. PIE system achieved a significantly higher classification efficacy than PCNE system (92.06% vs. 73.10%, *p* < 0.001). Regarding acceptability and feasibility, PIE system showed comparable performance to PCNE system (mean Likert score: 4.2 ± 0.83 vs. 3.7 ± 1.07, *p* = 0.113), with no statistically significant differences in coverage, clarity, and ease of use. Multivariate regression analyses identified physician decision-making errors (OR = 3.756, *p* < 0.001) and prescription/dispensing errors (OR = 3.139, *p* = 0.001) as two vital factors associated with successful DRPs classification in PIE system. Among classified DRPs, 77.24% were mild clinical impact (Level 1), 96.04% were related to prescription issues, and 95.80% of pharmacist interventions were accepted.

**Conclusion:**

PIE system, a modified DRPs classification system adapted to Chinese healthcare settings, was successfully established and validated. PIE system provides a valuable tool for DRPs management, source tracing, and pharmacist performance evaluation in China.

## 1 Introduction

Drug-related problems (DRPs) are defined as “events or circumstances involving drug therapy that actually or potentially interfere with desired health outcomes” (Pharmaceutical Care Network Europe, 2025), and they represent a critical barrier to effective pharmaceutical care. DRPs not only compromise patient safety and treatment efficacy but also lead to prolonged hospital stays, increased healthcare costs, and even mortality (Panagioti et al., 2019; Hodkinson et al., 2020; World Health Organization, 2024). Recent evidence indicates that medication-related harm continues to impose a substantial economic burden, with the WHO estimating the global cost of medication errors at about $42 billion annually, while a systematic review reported drug-related hospital admissions ranging from 1.3% to 41.3% across studies, with a large share being preventable (World Health Organization, 2017; Ayalew et al., 2019). In China, DRPs are likewise common in tertiary-hospital inpatient care: PCNE-based hospital studies have reported measurable DRP burdens ranging from approximately 0.25 DRPs per patient in neurology wards to >50% of patients experiencing DRPs in specialized surgical/cardiac units, with around 1 DRP per patient reported in some settings (Liu P. et al., 2021; Li et al., 2025; Gu et al., 2024). As clinical pharmacy services expand nationwide, Chinese tertiary hospitals increasingly require a DRP classification framework that is not only comprehensive but also operationally aligned with local prescription-review processes and documentation practices. However, direct application of the PCNE classification in Chinese hospital settings remains challenging. First, interpretations of several domains and terms may vary across departments, leading to ambiguity. Second, the system lacks sufficient granularity to adequately capture some high-frequency prescription review issues common in local practice. Third, its structure does not fully align with the “problem-source-intervention” workflow used by Chinese pharmacists, which often results in unclassifiable records or inconsistent coding in real-world applications. These practical gaps motivated us to develop a structured, China-adapted system (PIE) based on PCNE but optimized for tertiary-hospital implementation.

Over the past two decades, several DRP classification systems have been developed worldwide, including the Pharmaceutical Care Network Europe (PCNE) system, Westerlund system, Granada consensus, and DOCUMENT system (van Mil et al., 2004; Schaefer, 2002; Ibrahim et al., 2022; Williams et al., 2012). Among these, the PCNE system (currently version 9.1) is the most widely used internationally due to its comprehensive hierarchical structure, clear definitions, and ability to separate problems, causes, interventions, and outcomes (Pharmaceutical Care Network Europe, 2020). It has been validated in various healthcare settings across Europe, Asia, and Australia (Basger et al., 2014; Horvat and Kos, 2016; Alsayed et al., 2022). However, the application of PCNE V9.1 in Chinese tertiary hospitals faces significant challenges due to differences in healthcare systems, clinical practice patterns, pharmacist roles, and drug policies (Penm et al., 2014; Shanghai Hospital Association Clinical Pharmacy Management Professional CommitteeShanghai Pharmaceutical Association Hospital Pharmacy Professional Committee, 2022; Liu Q. et al., 2021). Clinical pharmacists in China have reported issues such as missing classification items, ambiguous terminology, and misalignment with domestic prescription review processes and pharmacist-prescriber communication models (Yun et al., 2023; Zheng et al., 2022). These limitations reduce the accuracy and efficiency of DRP classification, impeding effective source tracing and pharmacist performance management.

To address this gap, several countries have adapted the PCNE system to their local contexts. For example, Slovenia modified PCNE V6.2 to include potential problems as a subdomain, renamed “causes” to “risk factors,” and refined dispensing error categories, achieving high coding consistency (Horvat and Kos, 2016). Jordan and Belgium have also developed context-specific DRP classification systems with improved local applicability (Alsayed et al., 2022; Verheyen et al., 2024). In China, preliminary attempts to adjust existing classification systems have been made, but these efforts either lacked rigorous multi-center evaluation or failed to fully address the unique needs of Chinese tertiary hospitals (Penm et al., 2014; Liu Q. et al., 2021).

Against this background, we developed the Pharmacist Intervention Evaluation (PIE) System, a modified version of PCNE V9.1 tailored to the Chinese healthcare environment. The PIE system retains the core advantages of PCNE while introducing key modifications: (1) adding a primary category of “Severity Level” to quantify the clinical impact of DRPs; (2) splitting the PCNE “Causes” domain into “Prescription Issues” and “Source of Problems” to enhance traceability and align with domestic prescription management workflows; (3) refining and supplementing classification items to cover common DRPs in Chinese hospitals; and (4) optimizing terminology to reduce ambiguity in clinical practice.

## 2 Aim

The aim of this study was to develop and evaluate PIE system, a modified DRP classification derived from PCNE and tailored to Chinese tertiary hospitals, and to compare its classification efficacy, acceptability, and feasibility with the PCNE system in multi-center hospital practice; additionally, to explore factors associated with PIE classification efficacy using multivariable regression analysis.

## 3 Methods

### 3.1 Study design and setting

This study used a multi-phase methodological design comprising (1) development and content refinement of the PIE classification through structured expert consensus and pilot testing, and (2) a prospective, multi-center field evaluation using consecutively captured DRP reports from routine clinical pharmacy practice. This study conducted at two tertiary hospitals (Qilu Hospital central campus at Jinan city and the Qingdao campus at Qingdao city) between November 2023 and August 2025, and based on the opinion gathered from an expert panel and the assessment of patient scenarios conducted by clinical pharmacists. Neither the expert panel nor the clinical pharmacists are considered study participants; instead, they serve as specialized contributors to the establishment of the DRP classification framework. According to the Medical Ethics Committee of Qilu Hospital, ethical approval and written informed consent were not required for this study because the patient scenarios included in the research consist of both hypothetical cases and adapted, anonymized versions of clinical pharmacists’ routine classification records, and do not involve direct use of real patient data.

### 3.2 Development of the PIE system

**FIGURE 1:**
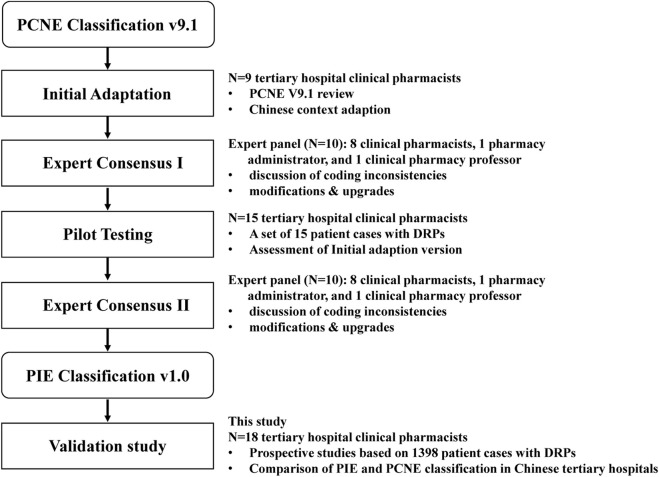
The multi-phase process to develop and validate the PIE v1.0 DRP classification system based on the PCNE classification v9.1.

The development of the PIE system followed a structured, multi-phase process (Figure 1), referencing the methodology used for adapting the PCNE system in previous international studies (Horvat and Kos, 2016; Verheyen et al., 2024).

Initial Adaptation: The research team (comprising 9 clinical pharmacy specialists with >5 years of experience) reviewed PCNE V9.1 and identified modifications needed for the Chinese context. Key adjustments included adding the “Severity Level” category, splitting “Causes” into “Prescription Issues” and “Source of Problems,” and refining items for drug selection, dosage, and administration routes based on Chinese clinical practice guidelines.

Expert Consensus I: An expert panel consisting of 10 members (7 clinical pharmacists from tertiary hospitals, 1 pharmacy administrator, and 1 clinical pharmacology professor) was convened to validate the content and structure of the initial PIE draft. The panel discussed each category and item, resolved discrepancies through iterative consultation, and formed the PIE classification system.

Pilot Testing: The PIE system was pilot-tested with 15 clinical cases (adapted from real-world DRPs in the study hospitals) by 5 clinical pharmacists. Feedback on clarity, usability, and completeness was collected, and minor adjustments were made to terminology and item descriptions.

Expert Consensus II: An expert panel consisting of 9 members (7 clinical pharmacists from tertiary hospitals, 1 pharmacy administrator, and 1 clinical pharmacology professor) was convened to validate the content and structure of the PIE draft. The panel discussed each category and item, resolved discrepancies through iterative consultation, and finalized the PIE v1.0 classification system.

The final PIE system consists of 6 primary categories, 27 secondary categories, and 83 tertiary categories (Table 1). The primary categories are: (1) Severity Level (L): quantifies the clinical impact of DRPs; (2) Prescription Issues (P): classifies problems related to drug selection, dosage form, administration, and course of treatment; (3) Source of Problems (R): identifies the origin of DRPs (e.g., physician decision-making, prescription/dispensing, patient adherence); (4) Intervention (I): describes pharmacist interventions targeting physicians, nurses, or patients; (5) Acceptance of Intervention (A): records whether the intervention was accepted and implemented; and (6) Outcome of DRPs (O): documents the resolution status of DRPs.

**TABLE 1:** Classification structure of the Pharmacist Intervention Evaluation (PIE) system (primary, secondary, and tertiary categories).

Primary category	Secondary category and code	Tertiary category and code
Severity level (L)	L0: No clinical impact	L0: No clinical impact
(Clinical impact of DRPs)	L1: Mild clinical impact	L1: Mild clinical impact (requires therapeutic intervention/intensified monitoring, no readmission/length of stay extension)
​	L2: Severe clinical impact	L2: Severe clinical impact (requires hospitalization/length of stay extension/permanent organ damage)
​	L3: Life-threatening	L3: Life-threatening
Prescription issues (P)	P1: Drug selection	P1.1: Unnecessary drug use (polypharmacy, no indication)
(Classification of prescription-related problems)	​	P1.2: Overtreatment (polypharmacy)
​	​	P1.3: Duplicate drug use (same pharmacological effect/active ingredient)
​	​	P1.4: Omitted necessary drug (undertreatment)
​	​	P1.5: Insufficient drug supply (undertreatment)
​	​	P1.6: Contraindicated drug use (wrong drug)
​	​	P1.7: Non-guideline-concordant drug (wrong drug)
​	​	P1.8: Non-optimal drug selection (wrong drug)
​	​	P1.9: Inappropriate drug combination (drug-drug interaction, incompatibility, improper solvent)
​	P2: Dosage form/specification	P2.1: Inappropriate dosage form/specification
​	P3: Dosage and administration	P3.1: Subtherapeutic dose
​	​	P3.2: Supratherapeutic dose
​	​	P3.3: Low drug reconstitution concentration
​	​	P3.4: High drug reconstitution concentration
​	​	P3.5: Insufficient administration frequency
​	​	P3.6: Excessive administration frequency
​	​	P3.7: Incorrect/unclear/missing administration time instructions
​	​	P3.8: Incorrect/unclear/missing administration route instructions
​	​	P3.9: Incorrect/unclear/missing administration sequence instructions
​	P4: Treatment course and precautions	P4.1: Too short treatment course
​	​	P4.2: Too long treatment course
​	​	P4.3: Unclear/missing special precautions
​	P5: Other issues	P5.1: Other situations (specify)
​	P6: No prescription issues	P6.1: No prescription issues
Source of problems (R)	R1: Physician decision	R1.1: Insufficient drug knowledge
(Tracing the origin of DRPs)	​	R1.2: Inadequate patient communication (insufficient information collection)
​	​	R1.3: Neglect of patient’s laboratory/examination results
​	​	R1.4: Neglect of patient’s current medication regimen
​	​	R1.5: Neglect of patient’s treatment progress and outcomes
​	​	R1.6: Other
​	R2: Prescription writing and dispensing	R2.1: Missing necessary prescription
​	​	R2.2: Prescription transcription errors/omissions
​	​	R2.3: Prescribed drug unavailable
​	​	R2.4: Dispensing errors (wrong drug, specification, or dose)
​	R3: Nursing execution	R3.1: Failure to administer drug
​	​	R3.2: Administration of wrong drug
​	​	R3.3: Dosage/administration errors (dose, concentration, frequency, time, sequence)
​	​	R3.4: Other execution errors (e.g., infusion rate, storage)
​	​	R3.5: Incorrect/unclear/missing medication counseling (交待)
​	R4: Patient use	R4.1: Patient’s failure to understand medication counseling
​	​	R4.2: Intentional non-adherence (missed doses/incorrect use)
​	​	R4.3: Unintentional non-adherence (missed doses/incorrect use despite understanding)
​	​	R4.4: Patient use of non-prescribed drugs
​	​	R4.5: Patient consumption of drug-interacting foods
​	​	R4.6: Improper drug storage by patient
​	​	R4.7: Patient’s inability to use drug/dosage form correctly
​	R5: Safety/efficacy evaluation	R5.1: Lack of/timely appropriate efficacy evaluation
​	​	R5.2: Lack of/timely appropriate safety evaluation
​	R6: Other	R6.1: Other causes (specify)
​	​	R6.2: No obvious cause
Intervention (I)	I0: No intervention	I0.1: No intervention
(Pharmacist interventions)	​	I0.2: No intervention (problem resolved spontaneously)
​	I1: Physician-level intervention	I1.1: Pharmacist identifies problem and informs physician
​	​	I1.2: Pharmacist identifies problem and provides solution to physician
​	​	I1.3: Pharmacist identifies problem and develops solution with physician
​	​	I1.4: Physician identifies problem and develops solution with pharmacist
​	I2: Nurse-level intervention	I2.1: Pharmacist identifies problem and informs nurse
​	​	I2.2: Pharmacist identifies problem and provides solution to nurse
​	​	I2.3: Pharmacist identifies problem and develops solution with nurse
​	​	I2.4: Nurse identifies problem and develops solution with pharmacist
​	I3: Patient-level intervention	I3.1: Pharmacist identifies problem and provides written materials only
​	​	I3.2: Pharmacist identifies problem and advises patient to consult physician
​	​	I3.3: Pharmacist identifies problem and consults with patient/family/caregiver
​	​	I3.4: Patient identifies problem and consults pharmacist (drug-related)
​	I4: Other interventions	I4.1: Other interventions (specify)
​	​	I4.2: Adverse drug event reporting to authorities
Acceptance of intervention (A)	A1: Intervention accepted	A1.1: Accept intervention and full implementation
(Acceptance of pharmacist interventions)	​	A1.2: Accept intervention, partial implementation (physician/nurse/patient)
​	​	A1.3: Accept intervention, no implementation
​	​	A1.4: Accept intervention, implementation status unknown
​	A2: Intervention rejected	A2.1: Reject intervention (not feasible)
​	​	A2.2: Reject intervention (disagreement by physician/nurse/patient)
​	​	A2.3: Reject intervention (other reasons, specify)
​	​	A2.4: Reject intervention (reason unknown)
​	A3: Other	A3.1: Intervention proposed, acceptance status unknown (no information)
​	​	A3.2: No intervention proposed
Outcome of DRPs (O)	O0: Outcome unknown	O0.1: Outcome of intervention unknown
(Resolution status of DRPs)	O1: Problem resolved	O1.1: Problem fully resolved
​	​	O1.2: Problem partially resolved
​	O2: Problem unresolved	O2.1: Problem unresolved (physician non-cooperation)
​	​	O2.2: Problem unresolved (nurse non-cooperation)
​	​	O2.3: Problem unresolved (patient non-cooperation)
​	​	O2.4: No need/possibility to resolve problem

### 3.3 Study participants

#### 3.3.1 Clinical pharmacists

Clinical pharmacists were eligible if they: (1) had ≥3 years of clinical pharmacy experience in tertiary hospitals; (2) were familiar with PCNE V9.1; (3) volunteered to participate, underwent standardized training, and completed training on the PCNE and PIE system. A total of 18 clinical pharmacists from 14 specialties were enrolled, with a mean clinical experience of 8.8 years (range: 3–20 years) (Table 2).

**TABLE 2:** Demographic and professional characteristics of participating clinical pharmacists.

Serial number	Gender	Specialty	Clinical pharmacy experience (Years)
1	Female	Oncology	18
2	Female	Respiratory and critical care	8
3	Male	Organ transplantation	5
4	Female	General surgery	8
5	Female	Rheumatology	5
6	Male	Pediatrics	9
7	Male	Intensive care unit (ICU)	13
8	Female	Cardiology	9
9	Female	Respiratory and critical care	8
10	Female	Gastroenterology	10
11	Female	Critical care medicine	3
12	Female	Obstetrics and gynecology	9
13	Male	Endocrinology	6
14	Female	Oncology	6
15	Male	Endocrinology	20
16	Female	Neurology	10
17	Male	Endocrinology	8
18	Male	Dermatology	3

#### 3.3.2 Patients

Patients were eligible if they: (1) were hospitalized at the study sites during the study period; (2) received pharmaceutical care from enrolled clinical pharmacists; (3) had complete medical records. Patients were excluded if they: (1) were transferred to other hospitals or departments during hospitalization; (2) had incomplete medical records; (3) refused follow-up. The sample size was calculated via PASS for the paired difference in DRP classification efficacy between PIE and PCNE V9.1 (α = 0.05, power = 0.80), assuming a 10% improvement with PIE, yielding a minimum requirement of ≥150 paired DRP records. We collected 1,398 paired DRPs through consecutive real-world sampling during the prespecified period, which exceeded the required sample size and therefore met the study requirements with adequate precision.

### 3.4 Data collection and classification

Enrolled clinical pharmacists identified DRPs during routine pharmaceutical care, including prescription review, ward rounds, patient counseling, and recorded details of each DRP using both PCNE V9.1 and the PIE system. For each DRP, pharmacists documented: (1) basic patient information (age, gender, diagnosis); (2) DRP-related details (severity, prescription issues, source, intervention, acceptance, outcome); (3) their classification experience (options: “Classifiable with high certainty,” “Classifiable with uncertainty,” “Unclassifiable”).

After data collection, a research assistant verified the completeness and accuracy of records. Discrepancies were resolved through consultation with the lead clinical pharmacist of the respective department.

### 3.5 Evaluation indicators

#### 3.5.1 Classification efficacy

Defined as the proportion of DRPs successfully classified by each system (i.e., pharmacists selected “Classifiable with high certainty” or “Classifiable with uncertainty”). The primary outcome was the difference in classification efficacy between PIE and PCNE systems.

#### 3.5.2 Internal consistency

To assess inter-rater reliability, clinical pharmacists classified 15 standardized cases (adapted from real DRPs) using both systems. Fleiss’ kappa (κ) was calculated for each primary category, with interpretation based on Landis and Koch’s criteria: ≤0.20 (poor), 0.21–0.40 (fair), 0.41–0.60 (moderate), 0.61–0.80 (substantial), and 0.81–1.00 (almost perfect) (Conger, 1980).

#### 3.5.3 Acceptability and feasibility

After data collection, pharmacists completed an 8-item questionnaire (5-point Likert scale: 1 = strongly disagree to 5 = strongly agree) evaluating: (1) DRP coverage; (2) clarity of categories; (3) ease of use; (4) suitability for inpatient DRP recording; (5) suitability for DRP summary; (6) ability to support source tracing; (7) overall satisfaction; (8) willingness to use in future practice.

#### 3.5.4 Factors influencing classification efficacy

Potential factors associated with successful classification using the PIE system, including hospital campus, department type (internal medicine vs. surgery), DRP occurrence time (during hospitalization, admission medication reconciliation, perioperative period, discharge medication), and source of DRPs (physician decision-making errors, prescription/dispensing errors, nursing execution errors, patient adherence errors), were further identified based on logistics regression algorithm.

### 3.6 Statistical analysis

Data were analyzed using SPSS 26.0 (IBM Corp., Armonk, NY, United States). Categorical variables were presented as counts and percentages, and continuous variables as mean ± standard deviation (SD). The classification efficacy of the two systems was compared using the paired χ^2^ test. Internal consistency was evaluated with Fleiss’ kappa. Acceptability and feasibility scores were compared using the Mann-Whitney U test. Univariate analysis (χ^2^ test or Fisher’s exact test) was used to screen factors associated with classification efficacy, and variables with *p* < 0.1 were included in multivariate binary logistic regression to identify independent factors. A two-tailed *p* < 0.05 was considered statistically significant.

## 4 Results

### 4.1 Characteristics of DRPs and study population

A total of 1,398 DRPs were identified and recorded during the study period, encompassing inpatients from two tertiary hospitals (Qilu Hospital central campus at Jinan city and the Qingdao campus at Qingdao city). The distribution of DRPs across departments was predominantly concentrated in internal medicine specialties (1,185/1,398, 84.76%), including cardiology, gastroenterology, respiratory medicine, and neurology, while surgical departments accounted for a smaller proportion (213/1,398, 15.23%). This discrepancy may reflect the higher complexity of medication regimens in internal medicine patients, containing polypharmacy for comorbidities like hypertension, diabetes, and cardiovascular disease, compared to surgical patients, who often have shorter hospital stays and more focused perioperative medication needs.

In terms of DRP occurrence time nodes, the majority (1,135/1,398, 81.18%) occurred during routine hospitalization, followed by discharge medication (128/1,398, 9.16%), perioperative periods (98/1,398, 7.01%), and admission medication reconciliation (37/1,398, 2.65%). The high proportion of DRPs during routine hospitalization highlights the need for continuous pharmaceutical care beyond initial admission and discharge, as ongoing adjustments to medication regimens present persistent risks. Discharge medication-related DRPs, though less frequent, remain clinically relevant given their potential to impact post-discharge adherence and outcomes. Patient demographics (age, gender, primary diagnosis) were comparable between the two hospitals, with no significant differences in DRP severity or type distribution, supporting the generalizability of the study results within tertiary hospital settings.

### 4.2 Internal consistency

**FIGURE 2:**
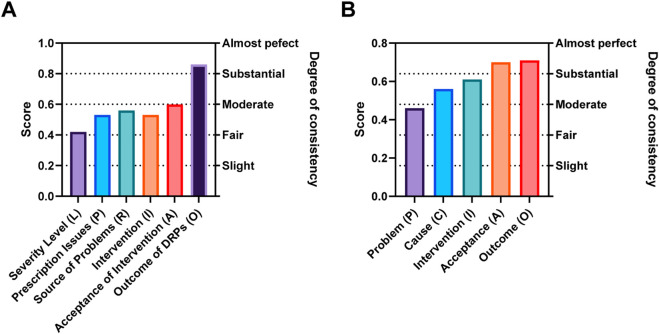
Inter-rater reliability (Fleiss’ kappa) of PIE System **(A)** and PCNE V9.1 **(B)**. The Fleiss’ kappa values were calculated for each primary category of the PIE System and PCNE V9.1, respectively, used to evaluate inter‐rater consistency. Consistency levels are defined according to Landis and Koch’s criteria: ≤0.20 (poor), 0.21–0.40 (fair), 0.41–0.60 (moderate), 0.61–0.80 (substantial), 0.81–1.00 (almost perfect).

Inter-rater reliability, measured by Fleiss’ kappa (κ), confirmed acceptable consistency for both the PIE System and PCNE V9.1 across all primary categories (κ ≥ 0.40), indicating that clinical pharmacists could apply both systems consistently (Figure 2). For the PIE System, four primary categories, “Severity Level” (κ = 0.42), “Prescription Issues” (κ = 0.53), “Source of Problems” (κ = 0.56), and “Intervention” (κ = 0.53), exhibited moderate consistency, while “Acceptance of Intervention” (κ = 0.60) reached substantial consistency, and “Outcome of DRPs” (κ = 0.86) achieved almost perfect consistency.

The moderate consistency observed for “Severity Level” is attributed to the inherent subjectivity of assessing clinical impact: pharmacists may vary in their judgment of whether a DRP requires “intensified monitoring” (Level 1) or constitutes “permanent organ damage” (Level 2), particularly for subclinical or potential DRPs. In contrast, “Outcome of DRPs” (fully resolved vs. unresolved) is a more objective endpoint, leading to near-perfect agreement. For PCNE V9.1, “Problem” (κ = 0.46) and “Cause” (κ = 0.56) showed moderate consistency, while “Intervention” (κ = 0.61), “Acceptance” (κ = 0.70), and “Outcome” (κ = 0.71) achieved substantial consistency. The slightly higher overall consistency of PCNE V9.1 (mean κ = 0.61 vs. PIE’s mean κ = 0.58) likely reflects the participating pharmacists’ prior familiarity with PCNE, as many had used earlier versions in clinical practice. Nevertheless, the PIE System’s consistency was still within the “moderate to substantial” range, confirming its reliability for routine use despite being a novel tool.

### 4.3 Classification efficacy

The PIE System demonstrated significantly higher classification efficacy than PCNE V9.1 (92.06% vs. 73.10%, *p* < 0.001), with 1,287 of 1,398 DRPs (88.84%) successfully classified by PIE compared to 1,022 of 1,398 (71.39%) by PCNE (Table 3). The 20.67% improvement in classification rate underscores the PIE System’s superior adaptability to Chinese clinical practice.

**TABLE 3:** Comparison of classification efficacy between PIE system and PCNE V9.1 (n = 1,398 DRPs).

Classification system	Classifiable (n)	Unclassifiable (n)	Classifiable rate (%)	Paired χ^2^	*p*-value
PIE system	1,287	111	92.06	174.61	<0.001*
PCNE V9.1	1,022	376	73.10	​	​

A DRP, was defined as “classifiable” if pharmacists selected “Classifiable with high certainty” or “Classifiable with uncertainty”; otherwise, it was “unclassifiable.” *p < 0.05 indicates statistical significance.

**FIGURE 3:**
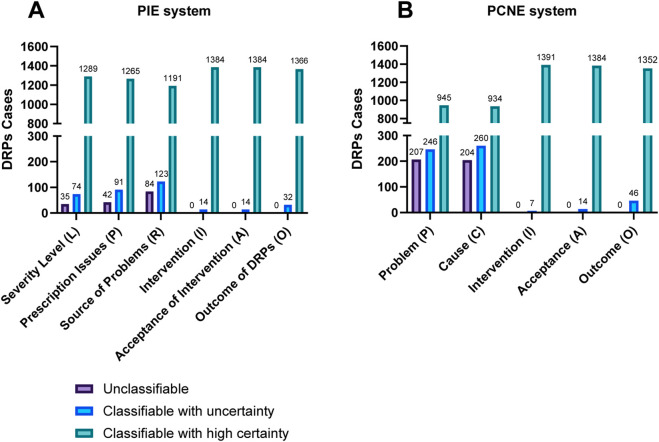
Comparison of classification efficacy between PIE System **(A)** and PCNE V9.1 **(B)** in first-level directory (N = 1,398 DRPs).

A breakdown of individual primary categories revealed the most pronounced differences in “Source of Problems” and “Prescription Issues” (Figure 3). For the PIE System, only 42 or 84 DRPs were unclassifiable across these two categories, compared to 204 unclassifiable cases for PCNE’s “Cause” domain. This discrepancy stems from the PIE System’s deliberate split of PCNE’s ambiguous “Cause” domain into two distinct categories, “Prescription Issues” and “Source of Problems”, which align with China’s national prescription review workflow and clarify the distinction between the nature of a prescription error (e.g., incorrect dosage) and its origin (physician decision-making vs. transcription error). Additionally, the PIE System’s refined terminology, for instance, “prescription transcription errors” instead of PCNE’s broad “dispensing error”, reduced ambiguity, particularly for common DRPs in Chinese hospitals such as medication reconciliation gaps and guideline-discordant prescribing.

Notably, PCNE V9.1 struggled to classify DRPs related to physician decision-making and prescription transcription, as these require context-specific categorization that the original system lacks. In contrast, the PIE System’s granular subcategories (e.g., R1.1: “insufficient drug knowledge” under physician decision) provided clear coding options, minimizing “unclassifiable” cases. These results confirm that the PIE System’s modifications address key limitations of PCNE V9.1 in capturing DRPs relevant to Chinese clinical practice.

### 4.4 Acceptability and feasibility

**FIGURE 4:**
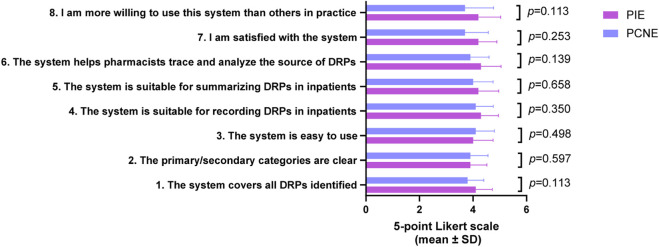
Pharmacists’ acceptability and feasibility scores of PIE System and PCNE V9.1 (5-point Likert scale, mean ± SD).

Pharmacists’ evaluations of acceptability and feasibility (Figure 4) showed that the PIE System outperformed PCNE V9.1 across all eight dimensions, though differences did not reach statistical significance (all *p* > 0.05). The PIE System scored highest in “ability to support source tracing” (4.3 ± 0.75 vs. PCNE’s 3.9 ± 0.70, *p* = 0.139) and “overall satisfaction” (4.2 ± 0.69 vs. 3.7 ± 0.87, *p* = 0.253), reflecting its core design goal of enabling DRP traceability, a critical requirement for quality improvement and pharmacist performance management in Chinese hospitals.

The only dimension where the PIE System scored marginally lower was “ease of use” (4.0 ± 0.75 vs. PCNE’s 4.1 ± 0.70, *p* = 0.498), which is attributed to its additional primary category (“Severity Level”) and more granular subcategories (83 tertiary categories vs. PCNE’s 60+). However, the negligible difference in scores indicates that pharmacists did not perceive this increased complexity as a significant barrier to use. Importantly, 83.3% of pharmacists reported a “willingness to use” the PIE System in future practice (score ≥4), compared to 66.7% for PCNE V9.1, highlighting the PIE System’s practical appeal.

Qualitative feedback from pharmacists emphasized that the PIE System’s ability to link DRPs to physician knowledge gaps and quantify severity aligned with hospital quality control initiatives, such as reducing medication errors and optimizing pharmacist-prescriber communication. While statistical significance was not achieved, the consistent trend of higher scores for the PIE System underscores its clinical relevance and potential for widespread adoption.

### 4.5 Factors influencing classification efficacy of PIE system

Univariate analysis identified four factors potentially associated with successful DRP classification using the PIE System: hospital campus (*p* < 0.001), department type (*p* = 0.051), DRP occurrence time (*p* < 0.001), physician decision-making errors (*p* = 0.009), and prescription/dispensing errors (*p* < 0.001) (Table 4). However, multivariate binary logistic regression confirmed only two independent predictive factors: physician decision-making errors (OR = 3.756, 95% CI: 2.234–6.316, *p* < 0.001) and prescription/dispensing errors (OR = 3.139, 95% CI: 1.831–5.383, *p* = 0.001) (Table 5).

**TABLE 4:** Univariate analysis of factors associated with successful DRP classification using PIE System (N = 1,398).

Factor	DRPs classifiable group (n = 1,287)	DRPs unclassifiable group (n = 111)	χ^2^ value	p-value
Hospital campus	​	​	16.393	<0.001*
- Central campus	1,135 (88.2)	83 (74.8)	​	​
- Qingdao campus	152 (11.8)	28 (25.2)	​	​
Department type	​	​	3.806	0.051*
- Internal medicine	1,098 (85.3)	87 (78.4)	​	​
- Surgery	189 (14.7)	24 (21.6)	​	​
DRP occurrence time	​	​	88.93	<0.001*
- During hospitalization	1,159 (90.0)	95 (85.6)	​	​
- Admission medication reconciliation	36 (2.8)	17 (15.3)	​	​
- Perioperative period	37 (2.9)	4 (3.6)	​	​
- Discharge medication	30 (2.3)	20 (18.0)	​	​
Physician decision errors	​	​	6.712	0.009*
- No	854 (66.4)	87 (78.4)	​	​
- Yes	433 (33.6)	24 (21.6)	​	​
Prescription/Dispensing errors	​	​	10.479	0.001*
- No	890 (69.2)	93 (83.8)	​	​
- Yes	397 (30.8)	18 (16.2)	​	​
Nursing execution errors	​	​	-	0.493
- No	1,264 (98.2)	108 (97.3)	​	​
- Yes	23 (1.8)	3 (2.7)	​	​
Patient adherence errors	​	​	-	0.120
- No	1,208 (93.9)	100 (90.0)	​	​
- Yes	79 (6.1)	11 (10.0)	​	​

**TABLE 5:** Multivariate binary logistic regression analysis of factors associated with classifiable DRPs cases using PIE system.

Variable	Reference group	β value	Standard error	Wald χ^2^	df	*p*-value	OR	95% confidence interval
Hospital campus	Central campus	−1.223	0.259	22.350	1	0.000	0.294	0.177–0.489
Department type	Internal medicine	−0.538	0.689	0.610	1	0.435	0.584	0.151–2.254
Physician decision errors	No	1.323	0.265	24.915	1	0.000	3.756	2.234–6.316
Prescription/Dispensing errors	No	1.144	0.275	17.282	1	0.000	3.139	1.831–5.383
DRP occurrence time	During hospitalization	​	​	4.844	3	0.184	-	-
- Admission medication reconciliation	During hospitalization	0.536	0.472	1.289	1	0.256	2.569	0.678–4.306
- Perioperative period	During hospitalization	−0.262	0.605	0.187	1	0.665	0.882	0.235–2.520
- Discharge medication	During hospitalization	0.292	0.968	0.091	1	0.763	0.401	0.201–8.922

Variables with *p* < 0.1 in univariate analysis were included. *p < 0.05 indicates statistical significance. “df” = degrees of freedom; “OR” = odds ratio.

The strong association between these two factors and successful classification is explained by their objective documentation and clear responsibility attribution. Physician decision-making errors, containing incorrect drug selection, dosage miscalculations, are captured in electronic prescriptions and pharmacy review logs, providing concrete evidence for coding. Similarly, prescription/dispensing errors, such as transcription mistakes, wrong dosage form dispensed, are tracked via hospital information systems and dispensing records, reducing subjectivity in classification. In contrast, DRPs related to nursing execution, including incorrect infusion rate, or patient adherence are often documented less systematically, leading to higher rates of unclassifiable cases.

The lack of statistical significance for hospital campus and department type in the multivariate model suggests that the PIE System’s classification efficacy is consistent across different settings within tertiary hospitals. This finding is reassuring, as it indicates the system’s generalizability regardless of geographic location or clinical specialty. Collectively, these results highlight the PIE System’s strength in capturing system-level DRPs (i.e., those originating from physician prescribing and pharmacy dispensing), which are priority targets for medication safety initiatives in China.

### 4.6 Characteristics of classified DRPs (PIE system)

Among the 1,287 DRPs successfully classified by the PIE System, detailed analysis revealed distinct patterns in severity, prescription issues, sources, interventions, acceptance, and outcomes, providing actionable insights for clinical practice.

#### 4.6.1 Severity level

**FIGURE 5:**
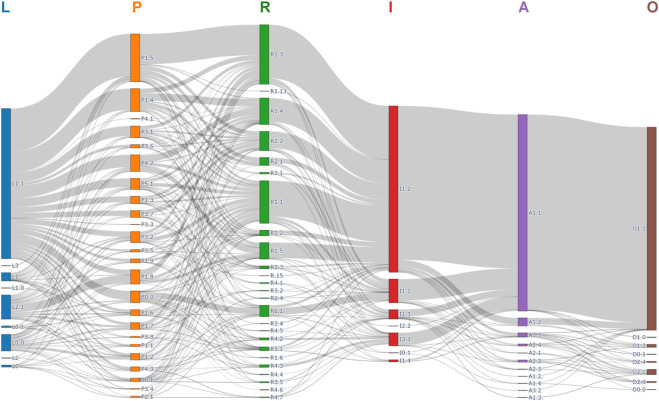
Sankey diagram of showing the distribution of primary and secondary category in PIE system (N = 1,287 classifiable DRPs). L: Severity Level, P: Prescription Issues, R: Source of Problems, I: Intervention, A: Acceptance of Intervention, O: Outcome of DRPs.

Most classified DRPs (994/1,287, 77.24%) were categorized as Level 1 (mild clinical impact), requiring therapeutic intervention or intensified monitoring but not leading to readmission or prolonged hospitalization (Figure 5). This was followed by Level 0 (no clinical impact, 223/1,287, 17.33%), Level 2 (severe clinical impact, 56/1,287, 4.35%), and Level 3 (life-threatening, 14/1,287, 1.09%). The high proportion of mild DRPs reflects the proactive role of clinical pharmacists in identifying and intervening early, before DRPs escalate to severe or life-threatening events. For example, dose adjustments for renally impaired patients (Level 1) and identification of potential drug-drug interactions (Level 0) are common interventions that prevent adverse outcomes. The low rate of severe/life-threatening DRPs (5.44%) further validates the effectiveness of pharmaceutical care in reducing medication-related harm in tertiary hospitals.

#### 4.6.2 Prescription issues

Nearly all classified DRPs (1,236/1,287, 96.04%) were associated with prescription problems, confirming that prescribing errors are the primary target for DRP prevention (Figure 5). Drug selection was the most frequent issue (751/1,287, 58.35%), with “failure to prescribe necessary drugs” (198/1,287, 15.38%) and “insufficient drug dosage” (182/1,287, 14.14%) being the top subcategories. These findings align with clinical practice, where gaps in guideline-concordant care, including omission of prophylactic anticoagulants in high-risk patients, and subtherapeutic dosing, such as inadequate antibiotic coverage, are common. Dosage and administration issues accounted for 25.02% (322/1,287) of DRPs, with “supratherapeutic dose” (109/1,287, 8.47%) and “incorrect administration frequency” (35/1,287, 2.8%) being prominent—likely due to physicians’ limited awareness of patient-specific factors, such as age, organ function, when adjusting regimens. Only 3.96% (51/1,287) of DRPs had no prescription issues, indicating that the PIE System effectively targets the root cause of most medication-related problems.

#### 4.6.3 Source of problems

Physician decision-making errors were the leading source of DRPs (976/1,287, 75.84%), followed by prescription/dispensing errors (198/1,287, 15.38%), nursing execution errors (65/1,287, 5.05%), patient use errors (32/1,287, 2.49%), and inadequate safety/efficacy evaluation (16/1,287, 1.24%) (Figure 5). Within physician decision-making errors, “insufficient drug knowledge” (328/1,287, 25.49%), “Neglect of patient’s laboratory/examination results” (211/1,287, 16.39%), and “inadequate consideration of current medication regimens” (184/1,287, 14.30%) were the most common subcategories. These results highlight the need for enhanced physician-pharmacist collaboration, such as pharmacist-led medication therapy management (MTM) and real-time drug information support. Prescription/dispensing errors were primarily driven by “transcription errors” (131/1,287, 10.18%), emphasizing the importance of electronic prescription systems and double-checking processes in pharmacies.

#### 4.6.4 Interventions

Pharmacist interventions were predominantly targeted at physicians (1,261/1,287, 97.98%), reflecting the hierarchical nature of medication decision-making in Chinese hospitals (Figure 5). The most common intervention was “providing direct solutions to physicians” (953/1,287, 73.97%), followed by “discussing solutions with physicians” (189/1,287, 14.70%) and “informing physicians of problems” (107/1,287, 8.31%). Only 14 and 8 DRPs (1.09% and 0.62%) involved interventions targeting nurses or patients, respectively, which is consistent with pharmacists’ primary role in prescribing optimization rather than direct patient counseling or nursing education in tertiary settings.

#### 4.6.5 Acceptance of interventions

The overall acceptance rate of pharmacist interventions was 95.80% (1,233/1,287), with 85.40% (1,099/1,287) fully implemented (Figure 5). Partial implementation accounted for 7.15% (92/1,287), while non-acceptance was rare (3.19%, 41/1,287). The primary reason for non-acceptance was physician disagreement (23/41, 56.10%), often due to conflicting clinical priorities, for example, a physician prioritizing a patient’s quality of life over guideline-concordant therapy for a chronic condition. Other reasons included “intervention not feasible” (7/41, 17.07%) and “reject intervention (reason unknown)” (11/41, 26.83%). The high acceptance rate (95.80%) underscores pharmacists’ credibility as medication experts. The low rejection rate (3.19%) also suggests minimal interprofessional conflict, which is critical for sustaining collaborative care models. Cases of physician disagreement highlight the need for structured communication frameworks, such as interdisciplinary rounds, to align pharmacist recommendations with clinical context.

#### 4.6.6 Outcome of DRPs

Most classified DRPs (1,142/1,287, 88.73%) were fully resolved, 3.34% (43/1,287) partially resolved, and 7.15% (92/1,287) unresolved (Figure 5). Unresolved cases were primarily due to physician non-cooperation (68/92, 73.91%) or patient non-adherence (17/92, 18.48%). The high-resolution rate (92.07% for full/partial resolution) demonstrates the PIE System’s utility in guiding effective interventions. For example, DRPs related to drug selection or dosage were almost always resolved through physician acceptance of pharmacist recommendations, while unresolved cases often involved non-modifiable factors (patient refusal to change medication). These outcomes confirm that the PIE System not only classifies DRPs but also supports targeted interventions that improve medication safety and effectiveness.

## 5 Discussion

This study developed and validated the PIE system, a modified PCNE V9.1 classification tailored to the Chinese healthcare environment, and demonstrated its superior classification efficacy compared with the original PCNE system in tertiary hospitals. The PIE system’s unique design, aligned with Chinese clinical practice, addresses key limitations of PCNE V9.1 and provides a standardized tool for DRP management and pharmacist performance evaluation.

The PIE system retains the hierarchical structure of PCNE but introduces critical adaptations that enhance its relevance to Chinese settings. First, the addition of the “Severity Level” category allows clinical pharmacists to quantify the clinical impact of DRPs, which is essential for prioritizing interventions and justifying pharmaceutical care resources—an important consideration in China’s resource-constrained tertiary hospitals (Qin et al., 2020). Second, splitting the PCNE “Causes” domain into “Prescription Issues” and “Source of Problems” improves traceability by separating the nature of the problem from its origin. This aligns with China’s national prescription review policy, which requires pharmacists to identify both prescription irregularities and their root causes (Rodrigues et al., 2019). Third, refining classification items to include context-specific issues (for example, traditional Chinese medicine-drug interactions, dosage form inappropriateness for elderly patients) addresses gaps in PCNE V9.1 that are particularly relevant in Chinese clinical practice (National Heal th Commission of the People’s Republic of China, 2023).

These modifications translated into significantly higher classification efficacy (92.06% vs. 73.10%), with fewer unclassifiable DRPs. The PIE system’s ability to capture nuanced DRP details—such as physician decision-making errors and prescription transcription issues—reflects its alignment with the workflow of Chinese clinical pharmacists, who play a central role in pre-prescription review and interprofessional communication (Gui et al., 2015).

The PIE system joins a growing body of context-adapted DRP classification systems worldwide. Similar to the Slovenian adaptation of PCNE V6.2 (Qu et al., 2019), the PIE system prioritizes practicality by simplifying ambiguous categories and adding context-relevant items. However, the PIE system goes further by integrating a severity grading system and separating prescription issues from problem sources, which are not features of the Slovenian system. Compared with the Belgian Be-CLIPSS system (Taegtmeyer et al., 2012), which focuses on hospital pharmacy activities, the PIE system is more comprehensive in DRP source tracing and intervention documentation, making it suitable for both clinical and administrative purposes.

The PIE system’s classification efficacy (92.06%) is comparable to or higher than other adapted systems. For example, the Slovenian system achieved 83% consistency for problems and 85% for risk factors (Horvat and Kos, 2016), while the Australian DOCUMENT system reported 82% inter-rater reliability (Williams et al., 2012). Differences between our findings and other national adaptations may reflect variations in PCNE versions, case sources, and rater training/familiarity, all of which can influence coding clarity and inter-rater agreement. In addition, PIE’s re-organization of source/issue domains is intended to reduce “unclassifiable” records and better match Chinese tertiary-hospital workflows, which may improve practical usability.

Multivariate regression identified physician decision-making errors and prescription/dispensing errors as key factors predicting successful classification. This is likely because these DRP sources have well-documented processes and clear responsibility attribution (physicians and pharmacists), making them easier to categorize (Taegtmeyer et al., 2012). In contrast, DRPs related to patient adherence or nursing execution are often influenced by subjective factors (e.g., patient-reported adherence, nursing workflow variations) that are harder to document systematically (Rodrigues et al., 2019). These findings highlight the PIE system’s strength in capturing system-level DRPs, which are a priority for quality improvement in Chinese hospitals (Ayeele and Tesfaye, 2021).

The PIE system’s high intervention acceptance rate (95.80%) and DRP resolution rate (92.07%) demonstrate its clinical value. By enabling precise source tracing, the PIE system helps pharmacists target interventions effectively—for example, focusing on physician education for drug selection errors or process optimization for prescription transcription errors. Additionally, the system’s structured documentation supports pharmacist performance management by quantifying intervention volume, acceptance rates, and resolution outcomes—an increasingly important requirement for clinical pharmacy departments in Chinese tertiary hospitals (Liu P. et al., 2021).

The PIE system also facilitates data-driven pharmaceutical care research. The detailed classification of DRPs allows for trend analysis, such as identifying high-risk departments or drug classes, and evaluating the impact of interventions over time (Westerlund et al., 1999). This aligns with China’s national strategy to promote evidence-based pharmaceutical care and improve medication safety (Ministry of Health of the People’s Republic of China, 2023).

While the PIE system did not show statistically significant differences in acceptability and feasibility compared with PCNE V9.1, it scored higher in key dimensions such as source tracing and overall satisfaction. The slightly lower score in “ease of use” may be due to the PIE system’s additional categories (6 vs. 5 primary categories), which require a short learning curve. However, the majority of pharmacists expressed willingness to use the PIE system in future practice, indicating that its benefits outweigh the minor increase in complexity.

## 6 Limitations

This study has several limitations. First, the sample size (1,398 DRPs) may not cover all DRP types encountered in Chinese tertiary hospitals, particularly rare or complex cases. Second, the study was conducted in two tertiary hospitals of a single university, which may limit generalizability to secondary hospitals or community pharmacies. Third, potential observer bias cannot be excluded because pharmacists were involved in both identifying DRPs and applying the PIE/PCNE classifications; familiarity with PCNE and expectations regarding PIE may have influenced coding behavior despite standardized training. Fourth, although data collection reflected routine practice, the same clinical teams contributed to classification and evaluation, and independent external coding in additional hospitals would further strengthen generalizability.

Future studies should expand the sample size to include more hospitals and healthcare settings, evaluate long-term reliability, and explore the PIE system’s utility for outpatient and community pharmacy DRPs. Additionally, developing a user manual and training materials could further improve the system’s ease of use and consistency.

## 7 Conclusion

The PIE system is a valid and reliable DRP classification tool adapted to the Chinese healthcare environment. It outperforms PCNE V9.1 in classification efficacy and shows comparable acceptability and feasibility in tertiary hospitals. The system’s unique design enhances DRP source tracing and supports pharmacist performance management, addressing critical needs in Chinese clinical pharmacy practice. Physician decision-making and prescription/dispensing processes are key factors influencing classification efficacy. The PIE system provides a standardized platform for DRP management and research, facilitating communication with international peers and contributing to the global advancement of pharmaceutical care.

## Data Availability

The original contributions presented in the study are included in the article/supplementary material, further inquiries can be directed to the corresponding authors.
